# Reply: Confounding in Mendelian randomisation studies

**DOI:** 10.1183/13993003.00995-2023

**Published:** 2023-07-20

**Authors:** Carl J. Reynolds, Cosetta Minelli

**Affiliations:** National Heart and Lung Institute, Imperial College London, London, UK

## Abstract

We thank J.J. Lawrence and R. Kumar for their comments, which express concerns regarding our findings of a causal effect of gastro-oesophageal reflux disease (GORD) on idiopathic pulmonary fibrosis (IPF) [1], as this gives us the opportunity to further discuss the issue of confounding in Mendelian randomisation (MR).

## Tweetable abstract

**Mendelian randomisation (MR) overcomes classical confounding issues that beset observational studies. “Horizontal” but not “vertical” pleiotropy can bias MR study findings but we found no evidence that it was present in our MR analysis of GORD on IPF.** https://bit.ly/3Jn7aeq

*Reply to J.J. Lawrence and R. Kumar*:

We thank J.J. Lawrence and R. Kumar for their comments, which express concerns regarding our findings of a causal effect of gastro-oesophageal reflux disease (GORD) on idiopathic pulmonary fibrosis (IPF) [1], as this gives us the opportunity to further discuss the issue of confounding in Mendelian randomisation (MR).

R. Kumar's example of spurious association between ice cream consumption and drowning risk resulting from confounding by high temperature illustrates the issue in observational studies when some confounders have not been adjusted for in the analyses, either because they have been disregarded, or because they have not been measured or are not suspected to be confounders. This is exactly why we chose to investigate whether GORD has a causal effect on IPF using the MR approach. With MR, the causal effect is estimated indirectly by looking at whether a genetic predisposition to GORD (GORD-associated genetic variants), which is present from birth and therefore not modified by classical confounders, increases IPF risk.

A form of confounding specific to MR, referred to as “horizontal” pleiotropy, could have biased our findings if some of the 59 variants used as proxies for GORD were also associated with other traits affecting IPF, but only if these traits acted through causal pathways independent from GORD. While we agree with J.J. Lawrence and R. Kumar on the dangers of pleiotropy in MR, we did not find any evidence that the association of some of our GORD variants with body mass index (BMI) and other traits may threaten the validity of our findings. We investigated this by: 1) assessing whether the causal effect estimates derived from each of the 59 GORD variants differed more than expected by chance, which would suggest the presence of pleiotropic variants among them, and they did not; and 2) repeating the analysis leaving out a variant at a time (including the FTO gene variant strongly associated with obesity), and the result did not change either. This reassured us against the possibility of spurious findings due to pleiotropy, but as a reply to a peer reviewer, we also repeated the analysis after excluding all GORD variants associated with obesity traits (data not shown), and again the results were similar. Regarding BMI in particular, it is possible that BMI may affect IPF through the same causal pathway as GORD; this, referred to as “vertical” pleiotropy, would not bias the MR results and, in fact, adjusting for BMI in this case would mask part (or all) of the effect of GORD on IPF.

## Shareable PDF

10.1183/13993003.00995-2023.Shareable1 This one-page PDF can be shared freely online.
Shareable PDF ERJ-00995-2023.Shareable

## References

[1] Reynolds CJ, Del Greco M F, Allen RJ, et al. The causal relationship between gastro-esophageal reflux disease and idiopathic pulmonary fibrosis: a bidirectional two-sample Mendelian randomization study. Eur Respir J 2023; 61: 2201585. doi:10.1183/13993003.01585-2022 37080571 PMC10209472

