# Supplementary material for: Reply: Confounding in Mendelian randomisation studies
Source: Eur Respir J. 2023 Jul 20;62(1):2300995. doi: 10.1183/13993003.00995-2023 (PMC10356965; doi:10.1183/13993003.00995-2023)
Supplement: Supplementary file 1 [file ERJ-00995-2023.Shareable.pdf]

SHAREABLE PDF

Reply: Confounding in Mendelian randomisation studies

Carl J. Reynolds and Cosetta Minelli

National Heart and Lung Institute, Imperial College London, London, UK.

Corresponding author: Carl J. Reynolds (carl.reynolds@imperial.ac.uk)

Shareable abstract (@ERSpublications)
Mendelian randomisation (MR) overcomes classical confounding issues that beset observational studies. "Horizontal" but not "vertical" pleiotropy can bias MR study findings but we found no evidence that it was present in our MR analysis of GORD on IPF.
https://bit.ly/3Jn7aeq

Cite this article as: Reynolds CJ, Minelli C. Reply: Confounding in Mendelian randomisation studies. *Eur Respir J* 2023; 62: 2300995 [DOI: 10.1183/13993003.00995-2023].

This single-page version can be shared freely online.

Copyright ©The authors 2023.

This version is distributed under the terms of the Creative Commons Attribution Licence 4.0.

Received: 13 June 2023
Accepted: 16 June 2023

Reply to J.J. Lawrence and R. Kumar:

We thank J.J. Lawrence and R. Kumar for their comments, which express concerns regarding our findings of a causal effect of gastro-oesophageal reflux disease (GORD) on idiopathic pulmonary fibrosis (IPF) [1], as this gives us the opportunity to further discuss the issue of confounding in Mendelian randomisation (MR).
